# Supplementary material for: Non-visual exploration of novel objects increases the levels of plasticity factors in the rat primary visual cortex
Source: PeerJ. 2018 Oct 23;6:e5678. doi: 10.7717/peerj.5678 (PMC6202959; doi:10.7717/peerj.5678)
Supplement: Table S1 — Counts of cells labeled for egr-1 and c-fos; densitometric measures of pCaMKII levels. [file peerj-06-5678-s001.docx]

| **Cell counts V1 - Egr-1** |  |  |
| --- | --- | --- |
|  |  |  |
| **Ctrl** | **1h** | **3h** |
|  |  |  |
| R03 Ctrl Lam 05 Sec02 | R04 1h Lam 02 Sec01 | R01 3h Lam01 Sec03 |
| 212 | 544 | 244 |
|  |  |  |
| R03 Ctrl Lam 05 Sec03 | R04 1h Lam02 Sec02 | R01 3h Lam 01 Sec05 |
| 194 | 474 | 127 |
|  |  |  |
| R03 Ctrl Lam 05 Sec04 | R04 1h Lam 02 Sec03 | R01 3h Lam 01 Sec04 |
| 203 | 427 | 315 |
|  |  |  |
| **R06 Ctrl Lam 04 Sec01** | **R05 1h Lam07 Sec02** | **R02 3h Lam02 Sec02** |
| **179** | **484** | **291** |
|  |  |  |
| **R06 Ctrl Lam 04 Sec04** | **R05 1h Lam07 Sec03** | **R02 3h Lam02 Sec01** |
| **207** | **477** | **212** |
|  |  |  |
| **R06 Ctrl Lam 04 Sec05** | **R05 1h Lam07 Sec06** | **R02 3h Lam02 Sec05** |
| **195** | **414** | **236** |
|  |  |  |
| R07 Ctrl Lam 03 Sec03 | R06 1h Lam04 Sec05 | R03 3h Lam03 Sec03 |
| 209 | 472 | 289 |
|  |  |  |
| R07 Ctrl Lam 03 Sec06 | R06 1h Lam04 Sec06 | R03 3h Lam03 Sec06 |
| 305 | 434 | 340 |
|  |  |  |
| R07 Ctrl Lam 03 Sec05 | R06 1h Lam04 Sec04 | R03 3h Lam03 Sec04 |
| 198 | 401 | 303 |
|  |  |  |
| **R08 Ctrl Lam 03 Sec04** | **R07 1h Lam 03 Sec01** | **R05 3h Lam 04 Sec01** |
| **185** | **457** | **272** |
|  |  |  |
| **R08 Ctrl Lam 03 Sec01** | **R07 1h Lam 03 Sec03** | **R05 3h Lam 04 Sec03** |
| **314** | **494** | **297** |
|  |  |  |
| **R08 Ctrl Lam 03 Sec03** | **R07 1h Lam 03 Sec04** | **R05 3h Lam 04 Sec04** |
| **264** | **531** | **202** |
|  |  |  |
| R09 Ctrl Lam02 Sec01 | R08 1h Lam 05 Sec04 | R06 3h Lam 03 Sec03 |
| 250 | 548 | 217 |
|  |  |  |
| R09 Ctrl Lam02 Sec06 | R08 1h Lam 04 Sec05 | R06 3h Lam 03 Sec04 |
| 311 | 472 | 218 |
|  |  |  |
| R09 Ctrl Lam02 Sec05 | R08 1h Lam 04 Sec06 | R06 3h Lam 03 Sec05 |
| 179 | 480 | 199 |

| **Cell counts V1 - c-Fos** |  |  |
| --- | --- | --- |
|  |  |  |
| **Ctrl** | **1h** | **3h** |
|  |  |  |
| R03 Ctrl Lam 07 Sec01 | R04 1h Lam 03 Sec02 | R01 3h Lam 02 Sec01 |
| 112 | 257 | 185 |
|  |  |  |
| R03 Ctrl Lam 07 Sec02 | R04 1h Lam 03 Sec01 | R01 3h Lam 02 Sec04 |
| 149 | 246 | 190 |
|  |  |  |
| R03 Ctrl Lam 07 Sec04 | R04 1h Lam 03 Sec03 | R01 3h Lam 02 Sec02 |
| 111 | 312 | 175 |
|  |  |  |
| **R06 Ctrl Lam 05 Sec01** | **R05 1h Lam 04 Sec06** | **R02 3h Lam 01 Sec01** |
| **128** | **278** | **162** |
|  |  |  |
| **R06 Ctrl Lam 05 Sec03** | **R05 1h Lam 04 Sec01** | **R02 3h Lam 01 Sec02** |
| **112** | **281** | **139** |
|  |  |  |
| **R06 Ctrl Lam 05 Sec04** | **R05 1h Lam 04 Sec03** | **R02 3h Lam 01 Sec04** |
| **153** | **238** | **187** |
|  |  |  |
| R07 Ctrl Lam 09 Sec03 | R06 1h Lam 06 Sec02 | R03 3h Lam 07 Sec01 |
| 112 | 242 | 148 |
|  |  |  |
| R07 Ctrl Lam 09 Sec02 | R06 1h Lam 06 Sec06 | R03 3h Lam 07 Sec02 |
| 149 | 315 | 177 |
|  |  |  |
| R07 Ctrl Lam 09 Sec06 | R06 1h Lam 06 Sec05 | R03 3h Lam 07 Sec03 |
| 111 | 207 | 161 |
|  |  |  |
| **R08 Ctrl Lam 07 Sec01** | **R07 1h Lam 02 Sec01** | **R05 3h Lam 01 Sec01** |
| **128** | **257** | **172** |
|  |  |  |
| **R08 Ctrl Lam 07 Sec02** | **R07 1h Lam 02 Sec02** | **R05 3h Lam 01 Sec02** |
| **112** | **291** | **148** |
|  |  |  |
| **R08 Ctrl Lam 07 Sec04** | **R07 1h Lam 06 Sec03** | **R05 3h Lam 01 Sec03** |
| **153** | **263** | **179** |
|  |  |  |
| R09 Ctrl Lam 07 Sec03 | R08 1h Lam 03 Sec03 | R06 3h Lam 04 Sec01 |
| 130 | 238 | 141 |
|  |  |  |
| R09 Ctrl Lam 07 Sec05 | R08 1h Lam 03 Sec05 | R06 3h Lam 04 Sec03 |
| 131 | 298 | 162 |
|  |  |  |
| R09 Ctrl Lam 07 Sec04 | R08 1h Lam 03 Sec06 | R06 3h Lam 04 Sec05 |
| 117 | 301 | 157 |

| **Densitometry - pCAMKII** | |  |  |
| --- | --- | --- | --- |
|  |  |  |  |
|  | **Ctrl** | **1h** | **3h** |
|  |  |  |  |
|  | R03 Ctrl Lam 04 Sec01 | R04 1h Lam 05 Sec02 | R01 3h Lam 03 Sec01 |
|  | 0,370 | 0,649 | 0,466 |
|  | 0,320 | 0,698 | 0,485 |
|  | 0,334 | 0,581 | 0,463 |
|  | 0,351 | 0,611 | 0,488 |
|  | 0,383 | 0,607 | 0,424 |
|  |  |  |  |
|  |  |  |  |
|  | R03 Ctrl Lam 04 Sec03 | R04 1h Lam 05 Sec04 | R01 3h Lam 03 Sec03 |
|  | 0,365 | 0,699 | 0,464 |
|  | 0,334 | 0,652 | 0,403 |
|  | 0,306 | 0,717 | 0,424 |
|  | 0,303 | 0,611 | 0,417 |
|  | 0,362 | 0,704 | 0,403 |
|  |  |  |  |
|  |  |  |  |
|  | R03 Ctrl Lam 04 Sec05 | R04 1h Lam 05 Sec06 | R01 3h Lam 02 Sec02 |
|  | 0,324 | 0,724 | 0,488 |
|  | 0,338 | 0,799 | 0,379 |
|  | 0,371 | 0,701 | 0,475 |
|  | 0,372 | 0,616 | 0,487 |
|  | 0,332 | 0,742 | 0,442 |
|  |  |  |  |
| mean | 0,344 | 0,674 | 0,447 |
|  | **R06 Ctrl Lam 02 Sec02** | **R05 1h Lam 05 Sec05** | **R02 3h Lam 04 Sec01** |
|  | 0,371 | 0,798 | 0,489 |
|  | 0,314 | 0,645 | 0,418 |
|  | 0,363 | 0,737 | 0,312 |
|  | 0,376 | 0,611 | 0,357 |
|  | 0,353 | 0,714 | 0,493 |
|  |  |  |  |
|  |  |  |  |
|  | **R06 Ctrl Lam 02 Sec03** | **R05 1h Lam 05 Sec04** | **R02 3h Lam 04 Sec04** |
|  | 0,366 | 0,764 | 0,499 |
|  | 0,385 | 0,703 | 0,479 |
|  | 0,363 | 0,724 | 0,465 |
|  | 0,388 | 0,717 | 0,482 |
|  | 0,324 | 0,703 | 0,402 |
|  |  |  |  |
|  |  |  |  |
|  | **R06 Ctrl Lam 02 Sec04** | **R05 1h Lam 05 Sec06** | **R02 3h Lam 04 Sec05** |
|  | 0,370 | 0,644 | 0,454 |
|  | 0,334 | 0,714 | 0,414 |
|  | 0,399 | 0,711 | 0,496 |
|  | 0,301 | 0,625 | 0,449 |
|  | 0,383 | 0,619 | 0,409 |
|  |  |  |  |
| mean | 0,359 | 0,695 | 0,441 |
|  | R07 Ctrl Lam 04 Sec04 | R06 1h Lam 03 Sec02 | R03 3h Lam 02 Sec02 |
|  | 0,365 | 0,664 | 0,441 |
|  | 0,371 | 0,643 | 0,422 |
|  | 0,372 | 0,683 | 0,452 |
|  | 0,363 | 0,678 | 0,425 |
|  | 0,388 | 0,630 | 0,432 |
|  |  |  |  |
|  |  |  |  |
|  | R07 Ctrl Lam 04 Sec05 | R06 1h Lam 03 Sec03 | R03 3h Lam 02 Sec04 |
|  | 0,324 | 0,670 | 0,404 |
|  | 0,338 | 0,609 | 0,479 |
|  | 0,385 | 0,664 | 0,443 |
|  | 0,316 | 0,628 | 0,449 |
|  | 0,332 | 0,613 | 0,408 |
|  |  |  |  |
|  |  |  |  |
|  | R07 Ctrl Lam 04 Sec06 | R06 1h Lam 03 Sec04 | R03 3h Lam 02 Sec05 |
|  | 0,371 | 0,699 | 0,446 |
|  | 0,366 | 0,788 | 0,421 |
|  | 0,334 | 0,698 | 0,467 |
|  | 0,306 | 0,687 | 0,427 |
|  | 0,353 | 0,622 | 0,430 |
|  |  |  |  |
| mean | 0,352 | 0,665 | 0,436 |
|  | **R08 Ctrl Lam 04 Sec01** | **R07 1h Lam 04 Sec01** | **R05 3h Lam 02 Sec02** |
|  | 0,384 | 0,661 | 0,430 |
|  | 0,363 | 0,605 | 0,421 |
|  | 0,353 | 0,619 | 0,474 |
|  | 0,362 | 0,642 | 0,402 |
|  | 0,324 | 0,642 | 0,448 |
|  |  |  |  |
|  |  |  |  |
|  | **R08 Ctrl Lam 04 Sec04** | **R07 1h Lam 04 Sec03** | **R05 3h Lam 02 Sec01** |
|  | 0,389 | 0,610 | 0,443 |
|  | 0,362 | 0,644 | 0,475 |
|  | 0,308 | 0,684 | 0,434 |
|  | 0,331 | 0,619 | 0,486 |
|  | 0,317 | 0,683 | 0,477 |
|  |  |  |  |
|  |  |  |  |
|  | **R08 Ctrl Lam 04 Sec05** | **R07 1h Lam 04 Sec04** | **R05 3h Lam 02 Sec03** |
|  | 0,384 | 0,602 | 0,489 |
|  | 0,334 | 0,676 | 0,511 |
|  | 0,306 | 0,689 | 0,476 |
|  | 0,353 | 0,657 | 0,463 |
|  | 0,332 | 0,625 | 0,496 |
|  |  |  |  |
| mean | 0,347 | 0,644 | 0,462 |
|  | R09 Ctrl Lam 04 Sec04 | R08 1h Lam 02 Sec03 | R06 3h Lam 02 Sec02 |
|  | 0,329 | 0,663 | 0,499 |
|  | 0,336 | 0,674 | 0,512 |
|  | 0,348 | 0,689 | 0,411 |
|  | 0,378 | 0,685 | 0,486 |
|  | 0,354 | 0,675 | 0,477 |
|  |  |  |  |
|  |  |  |  |
|  | R09 Ctrl Lam 04 Sec06 | R08 1h Lam 02 Sec03 | R06 3h Lam 02 Sec03 |
|  | 0,341 | 0,613 | 0,489 |
|  | 0,371 | 0,695 | 0,511 |
|  | 0,363 | 0,650 | 0,476 |
|  | 0,317 | 0,648 | 0,463 |
|  | 0,309 | 0,657 | 0,496 |
|  |  |  |  |
|  |  |  |  |
|  | R09 Ctrl Lam 04 Sec05 | R08 1h Lam 02 Sec03 | R06 3h Lam 02 Sec05 |
|  | 0,371 | 0,798 | 0,491 |
|  | 0,384 | 0,685 | 0,490 |
|  | 0,311 | 0,737 | 0,404 |
|  | 0,376 | 0,611 | 0,462 |
|  | 0,353 | 0,714 | 0,407 |
|  |  |  |  |
| mean | 0,349 | 0,680 | 0,472 |
